# Differential Dynamics of SARS-CoV-2 Binding and Functional Antibodies upon BNT162b2 Vaccine: A 6-Month Follow-Up

**DOI:** 10.3390/v14020312

**Published:** 2022-02-02

**Authors:** Giulia Matusali, Giuseppe Sberna, Silvia Meschi, Giulia Gramigna, Francesca Colavita, Daniele Lapa, Massimo Francalancia, Aurora Bettini, Maria R. Capobianchi, Vincenzo Puro, Concetta Castilletti, Francesco Vaia, Licia Bordi

**Affiliations:** 1 Laboratory of Virology and Biosafety Laboratories, National Institute for Infectious Diseases “Lazzaro Spallanzani” IRCCS, Via Portuense 292, 00149 Rome, Italy; giulia.matusali@inmi.it (G.M.); giuseppe.sberna@inmi.it (G.S.); giulia.gramigna@inmi.it (G.G.); francesca.colavita@inmi.it (F.C.); daniele.lapa@inmi.it (D.L.); massimo.francalancia@inmi.it (M.F.); aurora.bettini@inmi.it (A.B.); maria.capobianchi@inmi.it (M.R.C.); concetta.castilletti@inmi.it (C.C.); licia.bordi@inmi.it (L.B.); 2 Risk Management Unit, National Institute for Infectious Diseases “Lazzaro Spallanzani” IRCCS, Via Portuense 292, 00149 Rome, Italy; vincenzo.puro@inmi.it; 3 General Direction, National Institute for Infectious Diseases “Lazzaro Spallanzani” IRCCS, Via Portuense 292, 00149 Rome, Italy; francesco.vaia@inmi.it

**Keywords:** SARS-CoV-2, humoral response, anti-Spike IgG, neutralizing antibodies, correlates of protection

## Abstract

To investigate the dynamic association among binding and functional antibodies in health-care-workers receiving two doses of BNT162b2 mRNA COVID-19-vaccine, SARS-CoV-2 anti-RBD IgG, anti-Trimeric-S IgG, and neutralizing antibodies (Nabs) were measured in serum samples collected at 2 weeks, 3 months, and 6 months from full vaccination. Despite the high correlation, results for anti-RBD and anti-Trimeric S IgG were numerically different even after recalculation to BAU/mL following WHO standards indications. Moreover, after a peak response at 2 weeks, anti-RBD IgG levels showed a 4.5 and 13 fold decrease at 3 and 6 months, respectively, while the anti-Trimeric S IgG presented a less pronounced decay of 2.8 and 4.7 fold. Further different dynamics were observed for Nabs titers, resulting comparable at 3 and 6 months from vaccination. We also demonstrated that at NAbs titers ≥40, the area under the receiver operating characteristic curve and the optimal cutoff point decreased with time from vaccination for both anti-RBD and anti-Trimeric S IgG. The mutating relation among the anti-RBD IgG, anti-Trimeric S IgG, and neutralizing antibodies are indicative of antibody maturation upon vaccination. The lack of standardized laboratory procedures is one factor interfering with the definition of a correlate of protection from COVID-19.

## 1. Introduction

Nearly two years after the initial spread of SARS-CoV-2 infection, and one year from the start of the COVID-19 vaccination campaign, about 370 million people have been infected and more than 9.9 billion vaccine doses have been administered worldwide [1].

In this framework, a number of questions on the immune response elicited by either SARS-CoV-2 infection or COVID-19 vaccination have been answered while other remain open.

The current knowledge indicates that the different arms of the immune system (i.e., innate and adaptive cellular and humoral immunity) contribute in the control of infection [2], and that the induction of immune markers upon SARS-CoV-2 infection or vaccination may vary according to a number of factors including symptoms’ severity, age, gender, therapies intake, and vaccine type [3,4,5].

Although immune responses to infections are multifaceted, antibody based markers are often used as correlate of protection [6]. The advantage of binding antibodies markers lays on the fact that they are easy to titer, as often opposed to cellular immunity or neutralizing antibodies, and serological assays for high throughput platforms can be easily developed [7].

Nowadays, a correlate of protection for SARS-CoV-2 infection needs to be defined, nevertheless an increasing number of studies have been published on immune correlates’ analyses of SARS-CoV-2 vaccine [8,9,10,11], indicating that binding and neutralizing antibodies (NAbs) correlate with COVID-19 risk and vaccine efficacy.

In order to better understand the association among humoral response markers and protection from infection an effort in the harmonization of the results obtained from different laboratories worldwide is needed [12]. Indeed, in the current pandemic a large number of serological tests, based on different technologies and recognizing different epitopes of the S-protein, have been used along with neutralization assays. Even if an international standard for the evaluation of the antibody response to COVID-19 has been released by the WHO [13], its utility in enabling comparability of binding antibody tests has been criticized [14,15] and its use for standardization of neutralizing antibodies assays is still limited to few laboratories [13].

Moreover, we must consider that correlates of protection can vary in different populations (e.g., SARS-CoV-2 infected, COVID-19 vaccinees, or immunocompromised individuals) [9,16,17,18], for different vaccine formulation [19], upon the emergence of SARS-CoV-2 variants [20,21] and with time from vaccination or infection.

In this study, we analyzed the 6-month kinetics of anti-Spike antibodies, either binding to the receptor-binding domain (RBD) or directed against epitopes in the native trimeric spike protein, and of functional antibodies, measured by live SARS-CoV-2 microneutralization assays, in a population of BNT162b2 vaccinated health care workers (HCWs). The results obtained were analyzed to understand the dynamic association among the three different antibody markers in an effort to find predictive values of protective humoral response to a COVID-19 vaccine.

## 2. Materials and Methods

### 2.1. Study Cohort

A total of 156 serum samples were longitudinally collected from 52 HCW from the National Institute for Infectious Diseases “L. Spallanzani” who have been administered the BNT162b2 mRNA COVID-19 vaccine (Comirnaty, BioNTech Manufacturing GmbH, Mainz, Germany) during the period January–July 2021. Those subjects have been tested for anti-RBD IgG, anti-Trimeric Spike IgG, and SARS-CoV-2 neutralizing antibodies at 2 weeks (2w), 3 months (3mo), and 6 months (6mo) after the second dose of Comirnaty. The subjects had no previous history of SARS-CoV-2 infection, as self-reported or proven by the absence of positive SARS-CoV-2 molecular tests. Moreover, samples were tested for the presence of anti-Nucleocapsid IgG, in order to further identify and exclude possible natural infection. All serum samples tested were seronegative for the anti-N IgG.

The median age was 45.5 years [Interquartile-range (IQR) 33.0–53.8] (39 female). No active nor past SARS-CoV-2 infection were ever detected by molecular and serological assays or reported for any of the subjects included in the study.

### 2.2. SARS-CoV-2 Antibody Immunoassays

Two commercial assays were used to measure levels of anti-Spike SARS-CoV-2 IgG: (I) LIAISON^®^ SARS-CoV-2 TrimericS IgG assay, from DiaSorin S.p.A. (Saluggia, Italy), able to detect IgG against the spike viral protein in its native trimeric conformation, which includes the RBD and NTD sites from the three subunit S1, on LIAISON^®^ XL analyzer; (II) ARCHITECT SARS-CoV-2 IgG II Quantitative, by Abbott Laboratories (North Chicago, IL, USA), able to detect IgG against the Receptor Binding Domain (RBD), on Abbott ARCHITECT^®^ i2000sr analyzer (North Chicago, IL, USA).

After the release of a WHO standard preparation for SARS-CoV-2 binding antibodies [22], a conversion factor from Arbitrary Units/mL (AU/mL) became available for both assays and accordingly the results obtained in this study have been expressed in Binding Antibody Units/mL (BAU/mL) (DiaSorin: 1 BAU/mL = 2.6× AU/mL; Abbott: 1 BAU/mL = 0.142× AU/mL). For ARCHITECT SARS-CoV-2 IgG II positivity range spans from 50 AU/mL or 7.1 BAU/mL (positivity threshold) to 40,000 AU/mL, expanded to 80,000 AU/mL or 11,360 BAU/mL with an automated dilution; for LIAISON^®^ SARS-CoV-2 DiaSorin TriS IgG positivity range spans from 13 AU/mL or 33.8 BAU/mL to 800 AU/mL or 2080 BAU/mL, expanded to or 16,000 AU/mL or 41,600 BAU/mL following automated dilution.

Samples were also tested for the presence of anti-Nucleoprotein (anti-N) IgG, using Abbott ARCHITECT SARS-CoV-2 IgG assay.

### 2.3. SARS-CoV-2 Microneutralization Assay

Neutralizing antibodies were measured as previously described [23]. Briefly, seven two-fold serial dilutions (starting dilution 1:10) of heat-inactivated serum samples (56 °C for 30 min) were titrated in duplicate, mixed with medium containing 100 TCID50/well SARS-CoV-2 (SARS-CoV-2/Human/ITA/PAVIA10734/2020, clade G, D614G (S) Ref-SKU: 008V-04005, from EVAg portal) and incubated 30 min at 37 °C. Subsequently, sub-confluent Vero E6 cells seeded in 96-well tissue culture plates (ATCC, Manassas, VA, USA) were infected with 100 µL/well of virus/serum mixtures, and virus adsorption was carried out at 37 °C with 5% CO2 for 1 h at 37 °C. The serum/infection mix was removed and replaced with 10% fetal bovine serum (FBS; Cytiva HyClone; Marlborough, MA, USA) in Minimum essential Eagle’s medium (Sigma-Aldrich, St. Louis, MO, USA) and cytopathic effect (CPE) appearance was observed after 48 h. Neutralization titers were expressed as the reciprocal of the highest serum dilution inhibiting at least 90% of the CPE. When 90% inhibition of the CPE was not observed at the first dilution tested (1:10), the sample was considered not able to neutralize (neutralization titer < 10). Positive control samples showing medium neutralizing activity (i.e., neutralization titer = 40) were included in each assay, to standardize inter-assay procedures. Serum with known neutralization titer (Research reagent for anti-SARS-CoV-2 Ab NIBSC code 20/136) from the National Institute for Biological Standards and Control, Blanche Lane, Ridge, Herts, UK (NIBSC) was used as reference.

### 2.4. Statistical Analysis

Results were presented as median and IQR for continuous variables and geometric mean with 95% confidence interval (CI) for neutralizing antibodies (NAbs).

GraphPad Software (version 9.0.2) (La Jolla, CA, USA) was used to perform statistic comparisons (*p*-value < 0.05 was considered statistically significant).

Numerical outputs in BAU/mL obtained from the two anti-Spike IgG assays were compared using non-parametric Wilcoxon matched-pairs signed rank test. The correlation between antibody levels measured by different assays was evaluated using Spearman analysis. Differences in anti-RBD, anti-Trimeric S IgG, or NAb titers at different time points from vaccination were analyzed using non-parametric Friedman Dunn’s multiple comparison test.

Receiver operating characteristic curve (ROC) analysis was used to establish the optimal cutoff values for anti-RBD or anti-Trimeric S IgG to identify samples with NAb titer ≥ 40, using MedCalc Statistical Software version 19.2.6 (MedCalc Software bv, Ostend, Belgium; https://www.medcalc.org; accessed on 9 October 2021).

## 3. Results

### 3.1. Differential Kinetics of SARS-CoV2 Anti-RBD IgG and Anti-Trimeric S IgG

Two different high throughput serological assays, the first quantifying anti-RBD IgG, the second measuring anti-Trimeric S IgG were used in this study to investigate the nature and persistence of anti-Spike IgG elicited by COVID-19 vaccine in a population of HCWs.

Quantification of antibodies obtained by the two platforms were expressed in BAU/mL (according to manufacturer instructions, following indication by WHO), to establish if data from different assays could be considered interchangeable.

**Figure 1:**
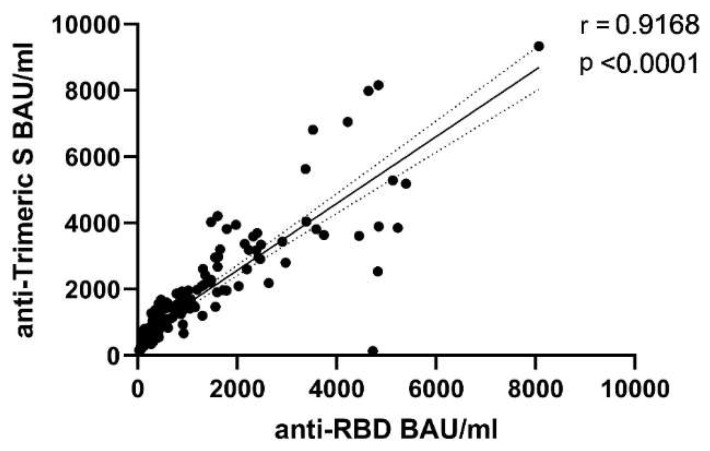
Correlation between SARS-CoV-2 anti-RBD IgG and anti-Trimeric S IgG antibodies. Results were expressed in BAU/mL. Spearman’s test was used for correlation analysis. Dotted lines represent 95% Confidence Interval (CI) of the best-fit line in linear regression analysis.

Values obtained with the two assays and compared using the non-parametric Wilcoxon matched-pairs signed-rank test resulted numerically different (*p* < 0.0001), thus confirming the difficulty in standardizing anti-Spike IgG measures produced by different laboratory platforms. Nevertheless, a very good correlation (*p* < 0.0001, r = 0.9168) between the two tests was observed, especially for values below 4000 BAU/mL (Figure 1).

**Figure 2:**
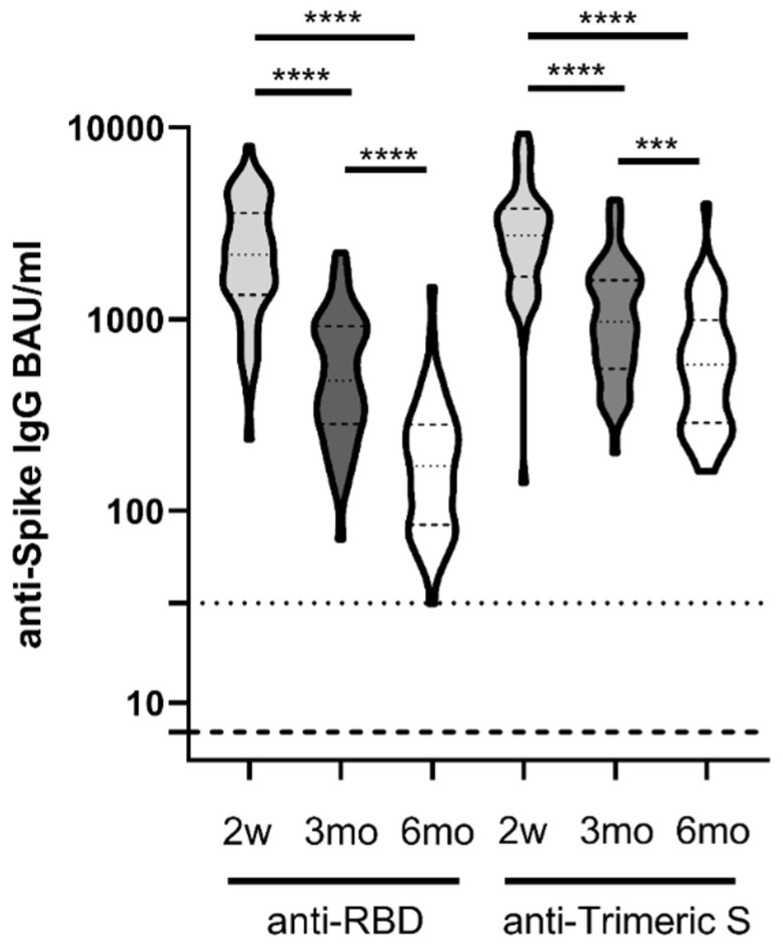
Patterns of anti-RBD IgG and anti-Trimeric S IgG persistence. Titers of anti-RBD IgG and anti-Trimeric S IgG antibodies in longitudinally collected samples from 52 HCWs after the second dose of Comirnaty (2w, 3mo, 6mo) are expressed as BAU/mL. Dot lines indicate limit of quantification for the two assays (33.8 BAU/mL for anti-TrimericS IgG and 7.1 BAU/mL for anti-RBD IgG). Friedman Dunn’s multiple comparison test was used for statistical analysis. *** *p* < 0.001; **** *p* < 0.0001.

Moreover, the analysis of anti-RBD and anti-Trimeric S IgG in the serum samples longitudinally collected after the second dose of BNT162b2 mRNA COVID-19 vaccine was performed (Figure 2).

As expected, the highest levels of anti-Spike IgG were measured by both assays at two weeks upon full vaccination (anti-RBD IgG median 2177, IQR 1343–3574; anti-Trimeric S IgG: median 2740, IQR 1671–3782). Subsequently, a significant reduction in IgG levels was observed at three months (anti-RBD IgG: median 479.7, IQR 283.7–920.8; anti-Trimeric S IgG: median 970.5, IQR 551.3–1598), further decreasing at six months (anti-RBD IgG: median 171.8, IQR 84.6–281.9; anti-Trimeric S IgG, median 578.5, IQR 288.5–991.0) (Figure 2). All serum samples showed detectable anti-Spike IgG at six months from COVID-19 vaccination.

Interestingly, we noticed a steeper decay for the anti-RBD IgG with respect to the anti-Trimeric S IgG (Figure 2): indeed a fold decrease of 4.5 (after 3 months) and 13 (after 6 months) of anti-RBD IgG levels was observed with respect to antibodies measured after 2 weeks. Differently, a 2.8 and 4.7 fold reduction in the anti-Trimeric S antibodies at 3mo and 6mo was revealed, respectively. These results also indicate an augmented divergence in the measurement of anti-RBD and anti-Trimeric S IgG as time from vaccination increased.

### 3.2. Comparative Kinetics of SARS-CoV-2 Neutralizing Antibodies and Spike-Binding IgG

To evaluate if the kinetics of neutralizing antibodies was comparable to the one observed for the anti-Spike IgG, we analyzed NAb titers in the serum samples derived from the 52 HCWs at 2w, 3mo, and 6mo after full vaccination.

**Figure 3:**
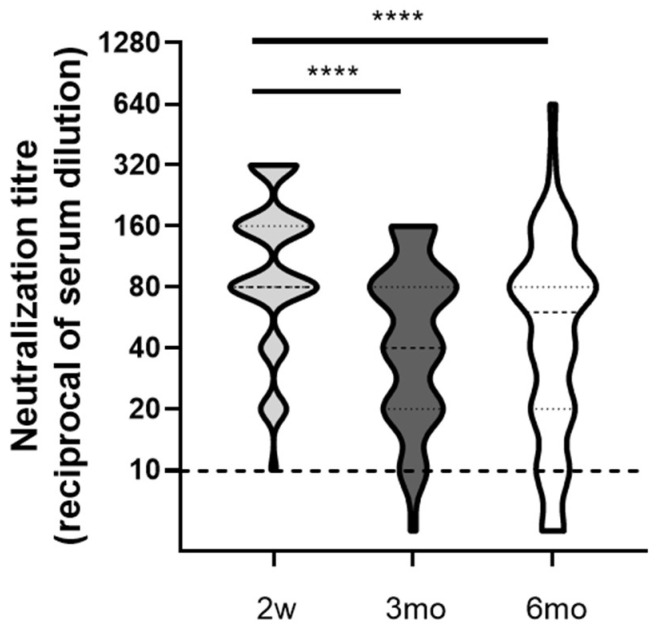
Neutralizing antibody titers in serum samples longitudinally collected (2w, 3mo, and 6mo). Neutralization titers are presented as reciprocal of serum dilution. Nab titer <10 is considered negative (not neutralizing). Dot line indicates the limit of Nab quantification (10). **** *p* < 0.0001.

High levels of NAbs were measured at 2w (Geo mean 97.71, CI 76.65–124.5), while decreased but comparable levels of NAbs at 3mo (Geo mean 44.50, CI 34.60–57.24) and 6mo (Geo mean 43.91, CI 31.89–60.46) were detected (Figure 3), thus suggesting a differential kinetics of functional antibodies as compared to anti-Spike IgG (both RBD or trimeric S specific).

It is noteworthy that, the neutralizing activity induced by the vaccine was lost (i.e., Neutralization titer < 10) in one individual at three months (Nab titer at 2w was 20) and three other individuals at 6 months from full vaccination. The Nab titers of the latter three HCWs were 80, 40, and 20 at 2 weeks, then 10, 40, and 20 at 3 months, respectively. For two of these HCWs, serum samples collected after a booster dose of COVID-19 vaccine were available and showed the restoration of neutralizing activity (NAb titer 160).

To assess the evolution of the relationship between measured anti-Spike IgG and NAbs at different time post full vaccination, correlation and regression operating characteristic curve (ROC) analyses were performed.

The correlation between anti-RBD and anti-Trimeric S IgG with NAb titers was highly significant (*p* < 0.0001) at each time point, being spearman r slightly variable over time (Table 1).

**Figure 4:**
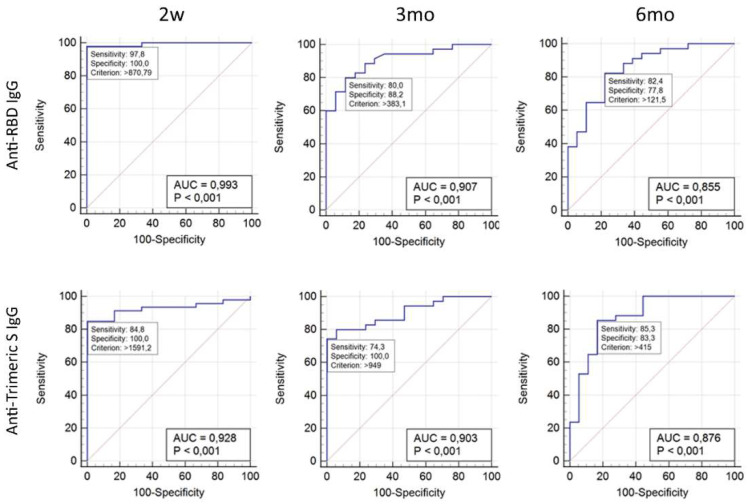
ROC analysis performed showing Anti-Trimeric S IgG and Anti-RBD antibody titers and their corresponding neutralization activity. ROC curves were obtained considering the antibody titers of the 52 individual samples measured at 2w, 3mo, and 6mo after the second dose of BNT162b2 mRNA COVID-19 vaccine. The curves were built using as cut-off a medium NAb titer ≥ 40.

The ROC built using as cut-off criterion a neutralizing antibody titers ≥ 40 (which corresponds to a medium level of neutralizing activity) showed an Area Under the Curve (AUC) of 0.99 and 0.93 for anti-RBD and anti-Trimeric S, respectively, when measured at the peak antibody response time point (2w). The AUC decreased at 3mo (0.907 for anti-RBD and 0.903 for anti-Trimeric S) and further reduced at 6mo (0.855 and 0.876) (Figure 4). The number of sera with neutralizing antibody titer ≥40 was 46 at 2w, decreasing at 3mo (35 sera) and at 6 mo (34 sera).

The optimal criterion, also decreased with time from vaccination from 870.8 (2 w) to 383.1 (3mo) and 121.5 (6mo) for anti-RBD and from 1591.2 (w) to 949.0 (3mo) and 415.5 (6mo) for the anti-Trimeric S (Figure 4 and Table 2). Accordingly, the criterion to have ≥99% of specificity consistently varied with time from the second dose of BNT162b2 mRNA COVID-19 Vaccine (Table 2), suggesting that different levels of anti-RBD or anti-Trimeric S are indicative of Nab titer ≥40 at different time points.

## 4. Discussion

In the ongoing COVID-19 vaccination era, quantitative serological assays are crucial for understanding the immunization status of the different population of vaccinated individuals; however, a further effort in the harmonization of the results obtained from different laboratories worldwide is needed, due to the lack of valuable standardization methods.

In this study, we observed the evolution of humoral response to BNT162b2 mRNA COVID-19 vaccine in 52 HCWs in a six-month follow-up study, by analyzing anti-Spike and functional antibodies persistence. Moreover, we compared the numerical output (expressed in BAU/mL) obtained by different widely used serological platforms trying to understand if a IgG threshold indicative of the presence of given titer of neutralizing antibodies could be established.

Consistent with previous report [15] our data reveal that, despite the high correlation, results for anti-RBD and anti-Trimeric S IgG were numerically different even after recalculation to BAU/mL following WHO standards indications. Importantly, we here show that time from vaccination may affect the relation among anti-RBD and anti-Trimeric S IgG. Indeed, after a peak response at two weeks, we observed a steeper decay for anti-RBD with respect to anti-Trimeric S IgG at both 3 and 6 months from the second COVID-19 vaccine dose. Further different dynamics were observed for neutralizing antibody titers which were comparable at 3 and 6 months from full vaccination. This observation is in line with a recent publication [24] but in contrast with two other studies showing a persistent decay in functional antibodies between the first and second trimester from mRNA COVID-19 vaccine administration [20,25].

The mutating relation among the anti-RBD IgG, anti-Trimeric S IgG, and Nabs, here observed, indicates that antibodies directed against both the RBD and other epitopes in the spike protein are responsible for blocking viral infection (e.g., the N-terminal domain NTD) [26]. It also suggests that the affinity of the antibodies to the spike viral protein induced by the Comirnaty vaccine evolves with time from vaccination [27], similarly to what observed upon SARS-CoV-2 infection [28].

The differential kinetics observed for anti-Spike IgG and functional antibodies clearly affected the threshold of IgG, that was directly correlated with neutralization activity. Indeed, the ROC analysis showed that the AUC and the optimal cutoff point decreased over time for both anti-RBD and anti-Trimeric S IgG. This indicates that lower levels of anti-Spike IgG can be associated with medium-to-high neutralization activity (i.e., Nabs titers ≥ 40) with time from vaccination, further suggesting antibodies’ affinity maturation. It is noteworthy that, recent studies on the definition of humoral correlates of protection upon COVID-19 vaccination did not explore this aspect [9,29].

Despite the size of sample tested and the higher number of females in the studied population could be considered limitations of our investigation, it is important to underline that the purpose of the study was to analyze the dynamic relation among humoral response markers. Notably, the same pattern of functional antibodies have been observed when analyzing a higher number (one-hundred) of serum samples from HCWs (unpublished data). Longer follow-up studies on the waning of humoral response and on the effect of booster doses are also ongoing in our hospital. Furthermore, preliminary results of experiments aimed at evaluating in vitro vaccine protection against SARS-CoV-2 variants of concern, are in line with published research articles showing a decay in the neutralization levels when using Delta or Omicron strains [30,31,32].

Overall, our report further emphasizes the urgency to harmonize results produced by different laboratories and define a proper correlate of protection against COVID-19, two actions of paramount importance for the management of the COVID-19 vaccination campaign in this pandemic era.

## Figures and Tables

**Table 1:** Correlation between anti-Spike IgG with NAb titers at three time points (Spearman r values are indicated).

Antibody Type	2w	3mo	6mo
Anti-RBD IgG	0.727	0.782	0.703
Anti-Trimeric S IgG	0.655	0.749	0.710

**Table 2:** Results of ROC analyses at 2 weeks, 3 months, and 6 months post full vaccination. Numbers indicate BAU/mL (Specificity-Sensitivity). The optimal criterion was calculated for a Nab titer ≥40.

	2w (Specificity-Sensitivity)	3mo (Specificity-Sensitivity)	6mo (Specificity-Sensitivity)
Anti-RBD Optimal criterion	870.8 (100.0–97.8)	383.1 (88.2–80.0)	121.5 (77.8–82.4)
Anti-Trimeric S Optimal criterion	1591.2 (100.0–84.8)	949.0 (100.0–74.3)	415.0 (83.3–85.3)
Anti-RBD criterion ≥99% of specificity	870.8 (100.0–97.8)	610.7 (100.0–60.0)	272.3 (100.0–38.2)
Anti-Trimeric S criterion ≥99% of specificity	1591.2 (100.0–84.8)	949.0 (100.0–74.3)	1280.3 (100.0–23.5)

## Data Availability

The data used and/or analyzed during the current study are available, only for sections non-infringing personal information, from the corresponding author on reasonable request.
